# Dual orexin receptor antagonists for the treatment of insomnia: systematic review and network meta-analysis

**DOI:** 10.1055/s-0043-1768667

**Published:** 2023-05-31

**Authors:** Rebeka Bustamante Rocha, Fernanda Ferreira Bomtempo, Gabriela Borges Nager, Giulia Isadora Cenci, João Paulo Mota Telles

**Affiliations:** 1 Universidade Federal do Amazonas, Faculdade de Medicina, Manaus AM, Brazil.; 2 Faculdade de Ciências Médicas de Minas Gerais, Faculdade de Medicina, Belo Horizonte MG, Brazil.; 3 Universidade Federal do Estado do Rio de Janeiro, Faculdade de Medicina, Rio de Janeiro RJ, Brazil.; 4 Faculdade Meridional, Faculdade de Medicina, Passo Fundo RS, Brazil.; 5 Universidade de São Paulo, Faculdade de Medicina, Departamento de Neurologia, São Paulo SP, Brazil.

**Keywords:** Orexin Receptor Antagonists, Sleep Initiation and Maintenance Disorders, Antagonistas dos Receptores de Orexina, Distúrbios do Início e da Manutenção do Sono

## Abstract

**Background** Several randomized clinical trials (RCTs) have shown that dual orexin receptor antagonists (DORAs) are effective in the treatment of chronic insomnia. However, the superiority of one particular DORA over the others remains unclear.

**Objective** To perform a network meta-analysis to evaluate the efficacy of different DORAs in patients with chronic insomnia.

**Methods** The Medline, Embase, and Cochrane Central databases were searched for RCTs that compared DORA with placebo in patients ≥ 18 years of age with a diagnosis of insomnia disorder. We pooled outcomes for wake time after sleep onset (WASO), latency to persistent sleep (LPS), total sleep time (TST), and adverse events (AEs).

**Results** We included 10 RCTs with 7,806 patients, 4,849 of whom received DORAs as the intervention. Overall, we found that DORAs were associated with the improvement of all analyzed efficacy outcomes. Concerning TST, an apparent dose-dependent pattern was noticed, with higher doses relating to a longer TST. Lemborexant 10mg provided the largest reduction in WASO (at month 1) in minutes (standardized mean difference [SMD] = -25.40; 95% confidence interval [95%CI] = -40.02–-10.78), followed by suvorexant 20/15mg (SMD = -25.29; 95%CI = -36.42–-14.15), which also appeared to provide the largest decrease in long-term WASO (SMD = -23.70; 95%CI = -35.89–-11.51). The most frequent AEs were somnolence, nasopharyngitis, and headache, with rates of up to 14.8%.

**Conclusion** Our results suggest that DORAs are associated with greater efficacy when compared with placebo in the treatment of insomnia, a complex 24-hour sleep disorder. Additionally, dosing might play an important role in the management of chronic insomnia.

## Resumo

**Antecedentes** Inúmeros ensaios clínicos randomizados (ECRs) têm demonstrado que os antagonistas duais do receptor de orexina ( *dual orexin receptor antagonists* , DORAs, em inglês) são eficazes no tratamento da insônia. Contudo, restam dúvidas quanto à superioridade de um DORA com relação aos outros.

**Objetivo** Realizar uma meta-análise em rede para avaliar a eficácia de diferentes DORAs em pacientes com insônia.

**Métodos** Foram feitas buscas nas bases de dados Medline, Embase e Cochrane Central por ECRs que comparassem DORAs e placebo em pacientes ≥ 18 anos de idade com diagnóstico de insônia. Os seguintes desfechos foram selecionados: tempo desperto após o início do sono ( *wake time after sleep onset* , WASO, em inglês), latência para o sono persistente ( *latency to persistent sleep* , LPS, em inglês), tempo total de sono ( *total sleep time* , TST, em inglês), e efeitos adversos (EAs).

**Resultados** Incluímos 10 ensaios clínicos com 7,806 pacientes, 4,849 dos quais receberam DORAs como intervenção. Os DORAs foram associados à melhoria de todos os desfechos de eficácia analisados. Em relação ao TST, um aparente padrão de dependência da dose foi identificado, com doses maiores se associando a um maior TST. Lemborexant 10 mg proporcionou a maior redução em WASO (no primeiro mês) em minutos (diferença padronizada das médias [ *standardized mean difference* , [SMD], em inglês) = -25.40; intervalo de confiança de 95% [IC95%] = -40.02–-10.78), seguido de suvorexant 20/15mg (SMD = -25.29; IC95% = -36.42–-14.15), o qual também proporcionou a maior diminuição em WASO no longo prazo (SMD = -23.70; IC95% = -35.89–-11.51). Os EAs mais frequentes foram sonolência, nasofaringite e cefaleia, com taxas de até 14.8%.

**Conclusão** Nossos resultados sugerem que os DORAs estão associados a uma maior eficácia quando comparados com placebo no tratamento da insônia, um complexo transtorno do sono de 24 horas. Além disso, a dosagem pode desempenhar um papel importante no manejo da insônia crônica.

## INTRODUCTION

Insomnia, which is defined as difficulty in sleep induction or maintenance along with daytime manifestations, is the most prevalent sleep disorder. 1 There are two main diagnostic classifications for this condition. According to the Diagnostic and Statistical Manual of Mental Disorders, 5th edition (DSM-5), 2 insomnia disorder can be of the short-term, recurrent, and persistent (symptoms lasting more than 3 months) types, whereas The International Classification of Sleep Disorders, Third Edition (ICSD-3), 1 categorizes it into short-term, chronic or other types.

Approximately 30% to 50% of adults experience short-term insomnia, and up to 10% meet the criteria for chronic insomnia, with a higher prevalence in older individuals. 3 This condition has been associated with a significant social burden, 4 and patients may have an increased risk of developing cardiovascular 5 6 and psychiatric diseases. 7 At present, the pharmacological treatment for insomnia still relies predominantly on GABA-modulating drugs such as benzodiazepines and Z-drugs. Nonetheless, they induce a global depression of the brain, and are linked to substantial side effects, potential risk of abuse, and rebound insomnia, which limit their long-term use. 8 9

In this context, dual orexin receptor antagonists (DORAs) emerged over a decade ago 10 with the prospect of being a safer option in the treatment of insomnia. Orexin is a neuropeptide that plays a role in the promotion of wakefulness, with two associated postsynaptic G-protein coupled receptors (orexin receptor 1, OxR1, and orexin receptor 2, OxR2). 11 12 The DORAs act by targeting the orexin cascade through the antagonism of both receptors, promoting sleep induction and maintenance, with a more localized effect. 13

Over the last years, several clinical trials were developed to assess the safety and efficacy of DORAs in both adults and elderly individuals, including suvorexant, 14 15 16 lemborexant, 17 18 filorexant, 19 almorexant, 20 and daridorexant. 21 22 The variety of available DORAs represents important therapeutic progress, but it also raises questions regarding the optimal dosing and the superiority of one particular DORA over the others. Therefore, we sought to perform an updated systematic review and network meta-analysis to evaluate the efficacy of different DORAs in patients with chronic insomnia.

## METHODS

### Eligibility criteria

Inclusion in the present meta-analysis was restricted to studies that met all of the following criteria: (1) randomized controlled trials (RCTs), (2) comparing DORAs with placebo or DORAs with placebo and zolpidem, (3) enrolling patients ≥ 18 years of age diagnosed with insomnia disorder according to DSM-4 or -5, (4) and reporting at least one of the outcomes of interest. We excluded studies with (1) crossover design, (2) overlapping patient populations, and (3) studies that used selective orexin receptor antagonists (SORAs) as the intervention.

Of note, most studies included compared DORAs with placebo, whereas some of them 20 21 23 added zolpidem as an active control drug ( Table 1 ). When available, we chose to include data regarding zolpidem to maximize the comparisons among different drugs. The patient, intervention, control, outcome, study design (PICOS) question and eligibility criteria are displayed in Table 2 .

**Table 1:** Baseline characteristics of the included studies

Study	Dual orexin receptor antagonist (DORA)	Insomnia disorder diagnostic criteria	Total number of patients (n)	Patient interventions/PLA (n)	Duration of the treatment	Age groups in years	Age in years: mean(± SD)	ISI, Mean(± SD)	Pooled outcomes	Sleep parameter assessment tool
Herring et al. 14 (2016): trial 1	SUV	DSM-4	1,021	SUV 20/15 mg (254); SUV 40/30 mg (383); PLA (384)	3 months	18–64; ≥ 65	SUV 20/15 mg:* 55(± 16); SUV 40/30 mg:* 56(± 15); PLA: 56(± 15)	SUV 20/15 mg:*: 16(± 4) SUV 40/30mg*: 16(± 4) PLA: 16(± 4)	WASO at month 1; WASO at month 3; LPS at month 1; TST at month 3 -	Electronic sleep diary; polysomnography
Herring et al. 14 (2016): trial 2	SUV	DSM-4	1,009	SUV 20/15 mg (239); SUV 40/30 mg (387); PLA (383)	3 months	18–64; ≥ 65	SUV 20/15 mg:* 56(± 16); SUV 40/30 mg:* 57(± 15); PLA: 57(± 15)	SUV 20/15mg*: 17(± 4) SUV 40/30mg*: 16(± 4) PLA: 16(± 4)		
Michelson et al. 15 (2014)	SUV	DSM-4	779	SUV 30/40 mg (521); PLA (258)	12 months	18–64; ≥ 65	SUV 30/40 mg:* 61.3(± 14.5); PLA: 62.0(± 14.6)	SUV 30/40mg*: 14.5(± 4.4) PLA: 16(± 4)	WASO at month 1; WASO at month 12; TST at month 12	
Black et al. 20 (2017)	ALM	DSM-4	709	ALM 100 mg (187); ALM 200 mg (177); ZOL 10 mg (168); PLA (177)	2 weeks	18–64	ALM 100 mg: 44.1; ALM 200 mg: 46.1; ZOL 10 mg: 45.1; PLA: 46.2	NA	Mean WASO at weeks 1 and 2; mean LPS at weeks 1 and 2	Electronic sleep diary; polysomnography
Fan et al. 16 (2017)	SUV	DSM-4	120	SUV 40 mg (60); PLA (60)	6 months	18–64	SUV 40 mg: 50.6(± 11.9); PLA: 51.4(± 12.2)	SUV 40mg: 14.3(± 4.0) PLA: 14.1(± 3.9)	TST at month 6	Electronic sleep diary
Rosenberg et al. 23 (2019): SUNRISE 1	LEM	DSM-5	1,006	LEM 5 mg (266);LEM 10 mg (269); ZOL 6.5 mg (263); PLA (208)	1 month	≥ 55 to < 65; ≥ 65	LEM 5 mg: 63.7(± 6.8); LEM 10 mg: 64.2(± 6.9); ZOL 6.5 mg: 64.3(± 7.1); PLA: 63.9(± 6.8)	LEM 5mg: 18.9(± 3.5) LEM 10mg: 19.0(± 3.3) ZOL 6.5mg 19.2(± 3.5) PLA: 19.4(± 3.6)	WASO at month 1 (nights 29 and 30)	Polysomnography
Dauvilliers et al. 21 (2019)	DAR	DSM-5	359	DAR 5 mg (60); DAR 10 mg (58); DAR 25 mg (60); DAR 50 mg (61); ZOL 10 mg (60); PLA (60)	1 month	18–64	DAR 5 mg: 42.4(± 11.4); DAR 10 mg: 45.2(± 10.9); DAR 25 mg: 46.4(± 11.9); DAR 50 mg: 45.0(± 11.5); ZOL 10 mg: 43.7(± 11.8); PLA: 45.7(± 10.4)	DAR 5mg: 20.8(± 2.6) DAR 10mg: 21.2(± 3.3) DAR 25mg: 21.3(± 2.7) DAR 50 mg: 21.1(± 2.7) ZOL 10 mg: 21.3(± 2.9) PLA: 21.3(± 2.7)	WASO at month 1 (week 4); LPS (days 1 and 2)	Electronic sleep diary; polysomnography
Kärppä et al. 18 (2020) SUNRISE 2	LEM	DSM-5	949	LEM 5 mg (316); LEM 10 mg (315); PLA (318)	6 months	< 65; ≥ 65 to < 75; ≥ 75	LEM 5mg: 54.2(± 13.7) LEM 10mg: 54.8(± 13.7) PLA: 54.5(± 14.0)	LEM 5 mg: 19.6(± 3.3); LEM 10 mg: 19.1(± 3.4); PLA: 19.0	WASO at month 6; TST at month 6	Electronic sleep diary
Mignot et al. 22 (2022): trial 1	DAR	DSM-5	930	DAR 25 mg (310); DAR 50 mg (310); PLA (310) (3.1)	3 months	18- 64; ≥ 65	DAR 25mg: 55.8(± 15.3) DAR 50mg: 55.5(± 15.3) PLA: 55.1(± 15.4)	DAR 25 mg: 19.0(± 4.3); DAR 50 mg: 19.3(± 4.0); PLA: 19.2(± 4.0)	WASO at month 1; WASO at month 3; LPS at month 1; TST at month 3 -	Electronic sleep diary; polysomnography
Mignot et al. 22 (2022): trial 2	DAR	DSM-5	924	DAR 10 mg (307); DAR 25 mg (309); PLA (308)	3 months	18- 64; ≥ 65	DAR 10 mg: 57.1(± 14.0); DAR 25 mg: 56.3(± 14.4); PLA: 56.7(± 14.1)	DAR 10 mg: 19.9(± 3.8); DAR 25 mg: 19.5(± 4.0); PLA: 19.6(± 4.1)		

Abbreviations: ALM, almorexant; DAR, daridorexant; DSM, Diagnostic and Statistical Manual of Mental Disorders; ISI, Insomnia Severity Index; LEM, lemborexant; LPS, latency to persistent sleep; NA, not available; PLA, placebo; SD, standard deviation; SUV, suvorexant; TST, total sleep time; WASO, wake time after sleep onset; ZOL, zolpidem.

Notes: *Dosing according to age group, with higher doses for non-elderly individuals and lower doses for elderly individuals; ISI: 0–7 = no clinically significant insomnia; 8–14 = subthreshold insomnia; 15–21 = clinical insomnia (moderate severity); 22–28 = clinical insomnia (severe).

**Table 2:** Patient, intervention, control, outcome, study design (PICOS) question and eligibility criteria

	Inclusion criteria	Exclusion criteria
**(P)** Patient	Insomnia disorder (according to the DSM-5) or primary insomnia (according to the DSM-4)	Overlapping patient populations
**(I)** Intervention	Dual orexin receptor antagonists (DORAs)	Selective orexin receptor antagonists (SORAs)
**(C)** Control	Placebo or placebo and zolpidem (active control drug)	–
**(O)** Outcome	Efficacy outcomes: wake time after sleep onset (WASO); latency to persistent sleep (LPS); total sleep time (TST)	–
**(S)** Study design	Randomized controlled trials (RCTs)	Studies not written in English; crossover design.

Abbreviation: DSM, Diagnostic and Statistical Manual of Mental Disorders.

### Search strategy and data extraction

We systematically searched the Medline, Embase, and Cochrane Central Register databases for RCTs published in English from inception to May 2022. The following search strategy was used: ( *Insomnia* OR *sleep initiation and maintenance disorders* ) AND ( *dual orexin receptor antagonist* OR *dual orexin receptor antagonists* OR *DORA* OR *DORAs* OR *orexin receptor antagonist* OR *orexin receptor antagonists* OR *ORA* OR *ORAs* OR *almorexant* OR *suvorexant* OR *lemborexant* OR *filorexant* OR *daridorexant* ). Previous systematic reviews, meta-analyses, as well as the references of the selected studies were also manually screened for any additional applicable studies. Two investigators (FFB and RBR) independently screened the search results using the Mendeley (Elsevier B.V., Amsterdam, The Netherlands) reference manager software and performed data extraction using the Microsoft Excel (Microsoft Corp., Redmond, WA, United States) software. Discrepancies were solved by the senior author (JPMT). The study protocol was registered in the International Prospective Register of Systematic Reviews 24 in May 2022 (PROSPERO: CRD42022334400).

### Endpoints

We extracted data from pre-specified efficacy outcomes, which included wake time after sleep onset (WASO), latency to persistent sleep (LPS), total sleep time (TST), and the most frequent adverse events (AEs). Depending on the protocol of each clinical trial, the mentioned outcomes could be presented as subjective measures, objective measures, or both. When both measures were available, objective measures were the preferred choice. Subjective (s) outcomes included sWASO and sTST, derived from electronic sleep diary entries made by patients. Polysomnography was performed for the objective outcomes.

### Quality assessment and certainty of evidence

Quality assessment and risk of bias were evaluated using the Cochrane Risk of Bias Assessment tool, version 2 (RoB-2, Cochrane, London, United Kingdom). Two independent authors (RBR and GBN) completed the risk of bias analyses according to five domains: randomization process, deviations from intended interventions, missing outcome data, measurement of outcome, and selection of reported results. The risk of bias was stratified into low risk of bias, high risk of bias, and some concerns. Disagreements were solved by the senior author (JPMT). Certainty of evidence was assessed using the Grading of Recommendations Assessment, Development, and Evaluation (GRADE) approach based on each efficacy outcome included in the present study (WASO, LPS, and TST).

### Statistical analysis

The present systematic review and meta-analysis followed the Cochrane Collaboration and the Preferred Reporting Items for Systematic Reviews and Meta-Analysis (PRISMA) statement guidelines. 16 The outcomes were compared using standardized mean differences (SMDs). The meta-analysis for WASO was performed according to the treatment period (1 month or ≥ 3 months). For LPS, data from up to one month was used, and TST analysis was made using the longest interval available from each study. Inconsistency was assessed using I-squared (I ^2^ ) statistic, and values > 50% were considered representative of substantial heterogeneity. Random-effects models were used, and all analyses were performed using the “netmeta” R package (R Foundation for Statistical Computing, Vienna, Austria). The R software (R Foundation for Statistical Computing) was used to perform statistical analysis.

## RESULTS

### Study selection and baseline characteristics

**Figure 1:**
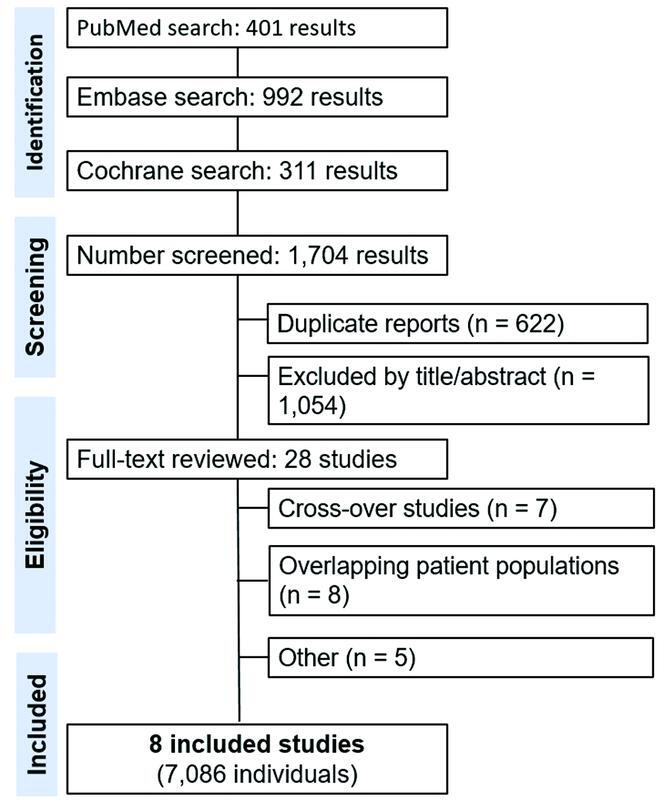
PRISMA flowchart of the screening and selection of studies.

The initial literature search yielded 1,704 results ( Figure 1 ). A total of 622 records were found to be duplicated, and 1,054 studies were excluded after reading the title/abstract. After full-text revision of the remaining 28 studies, 10 RCTs from 8 articles were included (2 articles 14 22 reported 2 RCTs each), accounting for 7,806 participants.

A total of 4,849 patients received a DORA as the intervention. Suvorexant was used in 1,844 patients, daridorexant, in 1,475, lemborexant, in 1,166, and almorexant, in 364 subjects. The follow-up ranged from 2 weeks to 12 months, and the drug doses ranged from 5 mg (lemborexant and daridorexant) to 200 mg (almorexant). Specifically, the doses of suvorexant ranged from 15 mg to 40 mg. The high doses (of 30 mg and 40 mg) included in these studies are not currently approved. In contrast, for daridorexant, the doses ranged from 5 mg to 50 mg. The lower doses (of 5 mg and 10 mg) are non-therapeutic, and not approved either. The main characteristics of the included studies are reported in Table 1 .

### Pooled analysis of all studies

Four network meta-analyses were conducted for 3 efficacy outcomes: (1) WASO month 1 and WASO ≥ 3 months, (2) LPS, and (3) TST.

### WASO month 1

**Figure 2:**
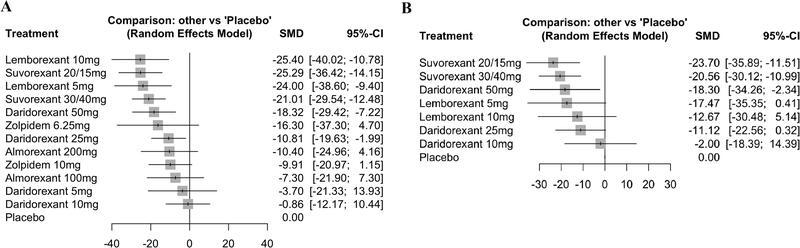
Wake time after sleep onset (WASO). WASO at months 1 ( **A** ) and 3 ( **B** ).

A total of six studies 14 15 17 20 21 22 (eight trials) contributed to this outcome. Figure 2A presents the forest plot of the treatment effect of the available DORAs compared with placebo. Negative values indicate a reduction in WASO and favor DORAs. No significant effects were observed for zolpidem 6.25 mg ( *p* = 0.13), zolpidem 10 mg ( *p* = 0.08), almorexant 100 mg ( *p* = 0.33), almorexant 200 mg ( *p* = 0.16), and lower doses of daridorexant: 5 mg ( *p* = 0.68), and 10 mg ( *p* = 0.88). Lemborexant and suvorexant had the largest reductions in WASO in minutes. Lemborexant 10 mg and 5 mg had very similar results (SMD =  -25.40 and -24.00 respectively), despite using a double dose. For daridorexant, the highest dose (of 50 mg) showed a greater effect (SMD = -18.32; 95% confidence interval [95%CI] = -29.42–-7.22). I ^2^ statistic tests revealed very high heterogeneity among the studies (tau-2 = 46.12; tau = 6.79; I ^2^ = 76.1% [95%CI = 54.2–87.5%]).

### WASO ≥ 3 months

Four studies 14 15 18 22 (six trials) evaluated this outcome ( Figure 2B ). The largest reductions in WASO ≥ 3 months were observed with suvorexant 20 mg/15 mg (SMD = -23.70; 95%CI = -35.89–-11.51), suvorexant 30 mg/40 mg (SMD = -20.56; 95%CI = -30.12–-10.99), and daridorexant 50 mg (SMD = -18.30; 95%CI = -34.26–-2.34). Lemborexant (in doses of 5 mg and 10mg) and lower doses of daridorexant (of 10 mg and 25 mg) did not show significant results. The I ^2^ statistic tests also revealed very high heterogeneity (tau-2 = 58.1; tau = 7.62; I ^2^ = 81.4% [95%CI = 56.8–92.0%]).

### LPS

**Figure 3:**
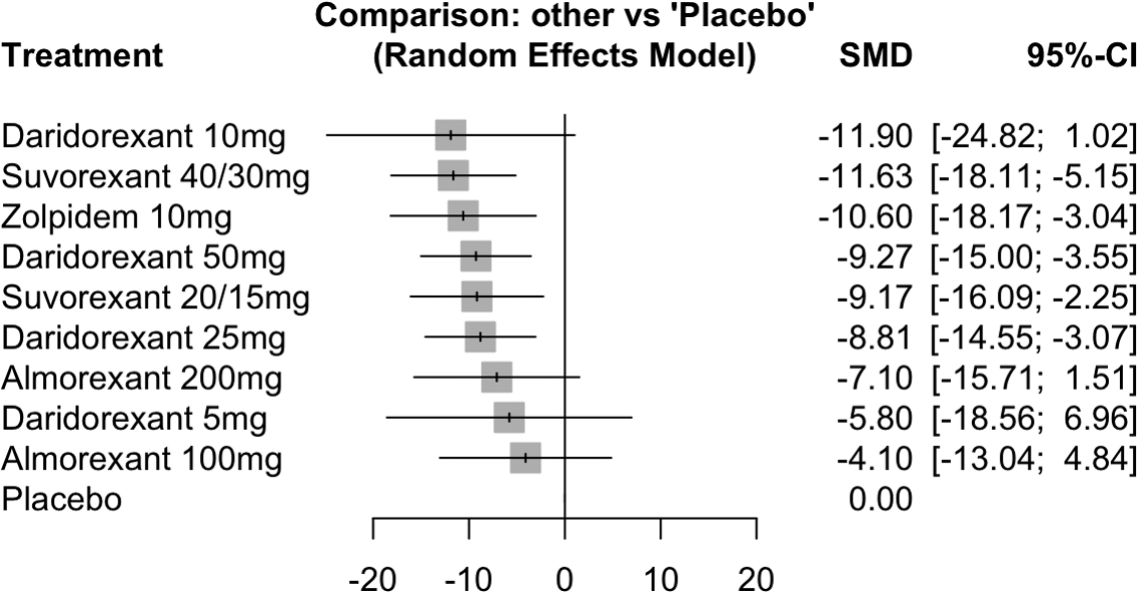
Latency to persistent sleep (LPS).

Four studies 14 20 21 22 (six trials) were included regarding this outcome. The results are shown in Figure 3 , with negative results indicating a reduction in LPS, thus favoring DORAs. Significant reductions in LPS were observed for high doses of suvorexant, of 40 mg/30 mg (SMD = -11.63; 95%CI = -18.17–-5.15), daridorexant 50 mg (SMD = -9.27; 95%CI = -15.00–-3.55), suvorexant 20 mg/15 mg (SMD = -9.27; 95%CI = -16.09–-2.25), and daridorexant 25 mg (SMD = -8.81; 95%CI = -14.55–-3.07). Almorexant (in doses of 100 mg and 200 mg) and daridorexant 5mg did not show significant results (95%CI includes 0). High heterogeneity was detected (tau-2 = 14.29; tau = 3.78; I ^2^ = 56.5% [95%CI = 4.2%–80.2%]) using I ^2^ statistic.

### TST

**Figure 4:**
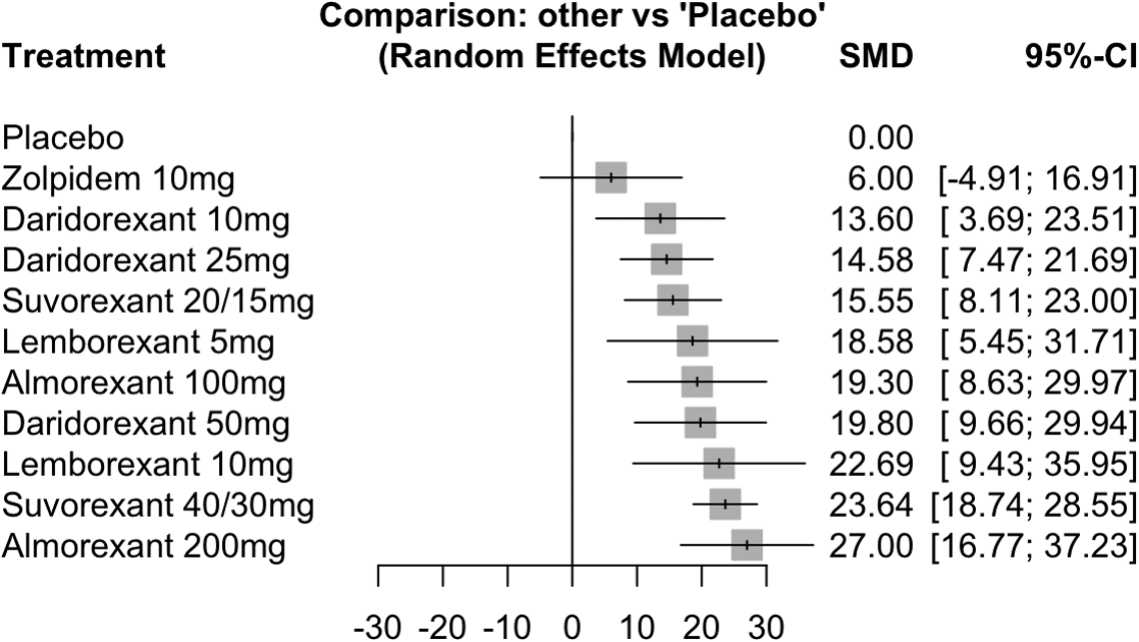
Total Sleep Time (TST).

Five studies (seven trials) 14 15 16 18 22 contributed to the meta-analysis of this outcome. The corresponding forest plot is displayed in Figure 4 , with positive SMD values indicating increases in TST, thus favoring DORAs. Significant results were observed for all DORAs, with increases in SMD values following what appears to be a dose-dependent pattern for each individual drug. The highest dose of almorexant (200 mg) showed a significant increase (SMD = 27.00; 95%CI = 16.77–37.23), as did suvorexant 40 mg/30 mg (SMD = 23.64; 95%CI = 18.74–28.55), lemborexant 10 mg (SMD = 22.69; 95%CI = 9.43–35.95), and daridorexant 50 mg (SMD = 19.80; 95%CI = 9.66–29.94). Apparently smaller but significant increases in TST were observed for lower doses of each DORA (daridorexant 10 mg/25 mg, suvorexant 20 mg/15 mg, lemborexant 5 mg, and almorexant 100 mg). No significant results were detected for zolpidem 10 mg (SMD = 6.0; 95%CI = -4.91–16.91). The I ^2^ statistic tests revealed low heterogeneity (tau-2 = 4.96; tau = 2.23; I ^2^ = 18.9% [0.0%; 63.7%]).

### AEs

The most frequent AEs were somnolence, nasopharyngitis, and headache ( Table 3 ), with incidence rates ranging from 4.1% to 13.2%, 4.0% to 12%, and 4.9% to 14.8% respectively. Somnolence appeared to be dose-dependent for both suvorexant and lemborexant. In contrast, the most common AEs in studies using daridorexant 21 22 were nasopharyngitis and headache.

**Table 3:** Most frequent adverse events in the included studies

Study	Drug	Most frequent AE: n/N (%)
Herring et al. 14 (2016): trial 1	SUV 20/15 mg; SUV 40/30 mg	Somnolence: 13/254 (5.1); **40/383 (10.7)**
	SUV 20/15 mg; SUV 40/30 mg	Nasopharyngitis: **18/254 (7.1)** ; 32/383 (8.4)
Herring et al. 14 (2016): trial 2	SUV 20/15 mg; SUV 40/30 mg	Somnolence: 20/239 (8.4); 40/387 (10.3)
Michelson et al. 15 (2014)	SUV 30/40 mg	Somnolence: 69/521 (13.2)
Black et al. 20 (2017)	ALM 100 mg; ALM 200 mg	Headache: 18/186 (9.7); 26/176 (14.8)
Fan et al. 16 (2017)	SUV 40 mg	Headache: 7/60 (11.7)
Rosenberg et al. 23 (2019): SUNRISE 1	LEM 5 mg; LEM 10 mg	Headache: **17/266 (6.4)** ; 13/268 (4.9)
	LEM 5 mg; LEM 10 mg	Somnolence: 11/266 (4.1); **19/268 (7.1)**
Dauvilliers et al. 21 (2019)	DAR 5 mg; DAR 10 mg; DAR 25 mg; DAR 50 mg	Headache: 6/60 (10); 5/58 (8.6); 5/60 (8.3); 5/61 (8.2)
Kärppä et al. 18 (2020) SUNRISE 2	LEM 5 mg; LEM 10 mg	Somnolence: 27/314 (8.6); 41/314 (13.1)
	LEM 5 mg; LEM 10 mg	Headache: 28/314 (8.9); 21/314 (6.7)
Mignot et al. 22 (2022): trial 1	DAR 25 mg; DAR 50 mg	Nasopharyngitis: 21/310 (7); 20/308 (6)
Mignot et al. 22 (2022): trial 2	DAR 25 mg; DAR 10 mg	Nasopharyngitis: 13/308 (4); 32/306 (12)

Abbreviations: AE, adverse event; ALM, almorexant; DAR, daridorexant; LEM, lemborexant; n, number of patients with an event; N, total number of patients; SUV, suvorexant.

Note: In some cases, the most reported adverse event differed depending on the dose of the same drug. Numbers in bold represent the most frequent AEs for that specific formulation.

### Quality assessment and certainty of evidence

**Figure 5:**
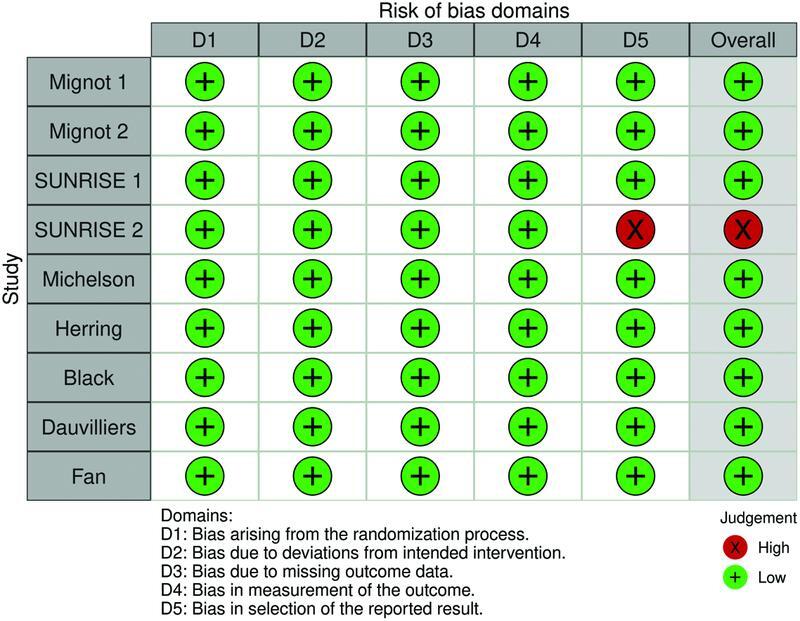
Risk of bias.

Overall, the studies were found to be of high quality. Only one study (Long-term Study of Lemborexant in Insomnia Disorder, SUNRISE 2) 18 had a high risk of bias, which arose from selective reporting ( Figure 5 ). Analyses of domains regarding outcome data were performed based on WASO. One study 16 did not contribute to the mentioned outcome, so TST was used.

According to the GRADE analysis, the quality of the evidence was rated as moderate for WASO (month 1 and ≥ 3 months). The quality of the evidence for LPS and TST was considered high. The downgrade in the certainty of evidence was due to inconsistency, which was stated as very serious, serious, and not serious for WASO, LPS and TST respectively ( Supplementary Material Supplementary Figure S1 and Supplementary Table S1 [online only]).

## DISCUSSION

In the present meta-analysis of 10 double-blinded RCTs including 7,086 patients, we compared 4 different DORAs (almorexant, daridorexant, lemborexant, and suvorexant) with placebo and zolpidem. Our findings can be summarized as follows: (a) DORAs were associated with an improvement in all analyzed efficacy outcomes; (b) lemborexant and suvorexant resulted in the greatest reductions in WASO month 1; (c) suvorexant 20 mg/15 mg and 30 mg/40 mg, and Daridorexant 50 mg, the greatest reductions in WASO ≥ 3 months; (d) suvorexant 40 mg/30 mg and 20 mg/15 mg, and Daridorexant 50 mg and 25 mg presented significant reductions in LPS; (e) regarding TST, significant results were observed for all DORAs, with an apparent dose-dependent pattern; and (f) the most frequent AEs were mild, with incidence rates of up to 14.8%.

Daridorexant is the most recent DORA, and it was first approved in early 2022. The recommended therapeutic doses include both 25-mg and 50-mg formulations. 25 According to our analysis, lower doses of daridorexant did not show significant results for WASO (month 1 and ≥ 3 months) or LPS, besides resulting in the smallest increases in TST. These findings were already anticipated, because lower doses (of 5 mg and 10 mg) of daridorexant were not show to be efficacious in different clinical trials 21 22 25 and are currently not approved. Inversely, the 25-mg dose was superior to placebo in all efficacy outcomes, except for WASO ≥ 3 months, whereas the 50-mg dose was superior to placebo in all endpoints.

Regarding TST, an apparent dose-dependent pattern was noticed for each individual drug, with higher doses correlating with longer TST in minutes. Although dosing proved to be an important aspect of TST, it was not the case for other outcomes. For WASO and LPS, such a clear distinction was not observed. Overall, lemborexant 5 mg and 10 mg presented very similar results, despite dose differences. It is also important to note that although higher doses of suvorexant are effective and outperform lower doses (of 15 mg/20 mg) for some endpoints (LPS and TST), they are not currently approved due to safety concerns. 26 At present, suvorexant is the only DORA recommended in the guidelines, with a maximum dosage of 20 mg a day. 3

Regarding the safety profile of DORAs, we observed predominantly mild AEs with low incidence rates (from 4.1% to 14.8% overall). The most common AEs were somnolence, nasopharyngitis, and headache. According to a previous meta-analysis by Xue et al., 27 DORAs were also associated with mild AEs, such as abnormal dreams, fatigue, and dry mouth. 17 This is another positive aspect of this drug class, since conventional sleep medications are consistently acknowledged for their negative side effects. Finally, a total of three DORAs are approved by the US Food and Drug Administration (suvorexant, lemborexant, and daridorexant), although their availability is still limited throughout the world.

### Limitations and strengths

The present study has limitations. First, the absence of direct comparisons regarding different DORAs. The data extracted for efficacy outcomes from all trials incorporated were restricted to differences between DORA and placebo, or Zolpidem and placebo. In addition, the studies only included adult individuals with insomnia disorder and excluded patients with important comorbidities. Therefore, the results of the present meta-analysis might not apply to all patients.

Finally, the tools to assess sleep parameters were heterogeneous. Most trials provided both subjective and objective outcome measures, as shown in Table 1 . Nonetheless, in most cases, polysomnography was only available for the primary outcomes, and additional outcomes were mainly derived from electronic sleep diary entries made by patients. Although these are important tools and are widely used by the scientific community, concerns regarding patient-related outcomes must be taken into consideration, especially because there is a high degree of variability and uncertainty in subjective patient evaluations of sleep. 28

Despite the known limitations, the present study has several strengths. It is the first to include two recently published trials on daridorexant, 22 the newest drug in this class, providing data from more than 1,800 patients. Moreover, the studies referenced in this meta-analysis were found to be of high quality, with 8 of 9 studies showing a low risk of bias.

In conclusion, our results suggest that DORAs are a safe class of drugs associated with greater efficacy when compared with placebo in the treatment of insomnia, which is a complex sleep-wake disorder. The pharmacological treatment for insomnia is challenging, and it must address several aspects of the disease, including sleep variables and next-day functioning. When using a DORA, dosing may play an important role in sleep maintenance, and higher doses correlate with longer TSTs for each individual drug. Additional trials comparing two or more DORAs are needed.

## References

[1] American Academy of Sleep Medicine * International Classification of Sleep Disorders 3 ^rd^ Edition *

[2] American Psychiatric Association American Psychiatric Association. DSM-5 Task Force *Diagnostic and Statistical Manual of Mental Disorders: DSM-5.* American Psychiatric Association 2013

[3] Sateia M J Buysse D J Krystal A D Neubauer D N Heald J L Clinical Practice Guideline for the Pharmacologic Treatment of Chronic Insomnia in Adults: An American Academy of Sleep Medicine Clinical Practice Guideline J Clin Sleep Med 2017 13 02 307 349 10.5664/jcsm.6470 27998379 PMC5263087

[4] DiBonaventura M Richard L Kumar M Forsythe A Flores N M Moline M The association between insomnia and insomnia treatment side effects on health status, work productivity, and healthcare resource use PLoS One 2015 10 10 e0137117 10.1371/journal.pone.0137117 26426805 PMC4591007

[5] Bathgate C J Edinger J D Wyatt J K Krystal A D Objective but not subjective short sleep duration associated with increased risk for hypertension in individuals with insomnia Sleep 2016 39 05 1037 1045 10.5665/sleep.5748 26951399 PMC4835301

[6] Bathgate C J Fernandez-Mendoza J Insomnia, Short Sleep Duration, and High Blood Pressure: Recent Evidence and Future Directions for the Prevention and Management of Hypertension Curr Hypertens Rep 2018 20 06 52 10.1007/s11906-018-0850-6 29779139

[7] Baglioni C Battagliese G Feige B Insomnia as a predictor of depression: a meta-analytic evaluation of longitudinal epidemiological studies J Affect Disord 2011 135(1-3):10 19 10.1016/J.JAD.2011.01.011 21300408

[8] Winrow C J Doran S M Gotter A L Pharmacological characterization of orexin receptor antagonists Sleep (Basel) 2011 34 A5 https://www.embase.com/search/results?subaction=viewrecord&id=L71510263&from=export

[9] Pałasz A Lapray D Peyron C Dual orexin receptor antagonists - promising agents in the treatment of sleep disorders Int J Neuropsychopharmacol 2014 17 01 157 168 23702225 10.1017/S1461145713000552

[10] Brisbare-Roch C Dingemanse J Koberstein R Promotion of sleep by targeting the orexin system in rats, dogs and humans Nat Med 2007 13 02 150 155 10.1038/NM1544 17259994

[11] Wang Z J Liu J F The Molecular Basis of Insomnia: Implication for Therapeutic Approaches Drug Dev Res 2016 77 08 427 436 10.1002/ddr.21338 27594319

[12] Winrow C J Turek F W Renger J J Genetic approaches for target identification in sleep/wake systems IDrugs 2008 11 11 811 816 https://www.embase.com/search/results?subaction=viewrecord&id=L352630042&from=export 18988125

[13] Morin C M Drake C L Harvey A G Insomnia disorder Nat Rev Dis Primers 2015 1 15026 10.1038/NRDP.2015.26 27189779

[14] Herring W J Connor K M Ivgy-May N Suvorexant in Patients With Insomnia: Results From Two 3-Month Randomized Controlled Clinical Trials Biol Psychiatry 2016 79 02 136 148 10.1016/j.biopsych.2014.10.003 25526970

[15] Michelson D Snyder E Paradis E Safety and efficacy of suvorexant during 1-year treatment of insomnia with subsequent abrupt treatment discontinuation: a phase 3 randomised, double-blind, placebo-controlled trial Lancet Neurol 2014 13 05 461 471 24680372 10.1016/S1474-4422(14)70053-5

[16] Fan B Kang J He Y Hao M Du W Ma S Efficacy and safety of suvorexant for the treatment of primary insomnia among Chinese: a 6-month randomized double-blind controlled study Neurol Asia 2017 22 01 41 47 https://www.cochranelibrary.com/central/doi/10.1002/central/CN-01365366/full

[17] Rosenberg R Murphy P Chou C Dhadda S Zammit G Moline M Comparison of lemborexant with zolpidem extended release and placebo: topline results from a phase 3 study in subjects 55 years and older with insomnia J Sleep Res 2018 27 16528880425

[18] Kärppä M Yardley J Pinner K Long-term efficacy and tolerability of lemborexant compared with placebo in adults with insomnia disorder: results from the phase 3 randomized clinical trial SUNRISE 2 Sleep 2020 43 09 zsaa123 10.1093/sleep/zsaa123 32585700 PMC7487867

[19] Connor K M Mahoney E Jackson S A Phase II Dose-Ranging Study Evaluating the Efficacy and Safety of the Orexin Receptor Antagonist Filorexant (MK-6096) in Patients with Primary Insomnia Int J Neuropsychopharmacol 2016 19 08 pyw022 10.1093/ijnp/pyw022 26979830 PMC5006195

[20] Black J Pillar G Hedner J Efficacy and safety of almorexant in adult chronic insomnia: a randomized placebo-controlled trial with an active reference Sleep Med 2017 36 86 94 10.1016/j.sleep.2017.05.009 28735928

[21] Dauvilliers Y Zammit G Fietze I A novel dual orexin receptor antagonist (act-541468) to treat insomnia: a randomized, double-blind, placebo-controlled, activere ference phase 2 study Sleep (Basel) 2019 42 A152 A153 10.1093/sleep/zsz067.374

[22] investigators Mignot E Mayleben D Fietze I Safety and efficacy of daridorexant in patients with insomnia disorder: results from two multicentre, randomised, double-blind, placebo-controlled, phase 3 trials Lancet Neurol 2022 21 02 125 139 35065036 10.1016/S1474-4422(21)00436-1

[23] Rosenberg R Murphy P Zammit G Comparison of Lemborexant With Placebo and Zolpidem Tartrate Extended Release for the Treatment of Older Adults With Insomnia Disorder: A Phase 3 Randomized Clinical Trial JAMA Netw Open 2019 2 12 e1918254 10.1001/jamanetworkopen.2019.18254 31880796 PMC6991236

[24] Page M J McKenzie J E Bossuyt P M The PRISMA 2020 statement: an updated guideline for reporting systematic reviews BMJ 2021 372 n71 10.1136/BMJ.N71 33782057 PMC8005924

[25] Markham A Daridorexant: First Approval Drugs 2022 82 05 601 607 10.1007/s40265-022-01699-y 35298826 PMC9042981

[26] Riemann D Baglioni C Bassetti C European guideline for the diagnosis and treatment of insomnia J Sleep Res 2017 26 06 675 700 10.1111/jsr.12594 28875581

[27] Xue T Wu X Chen S The efficacy and safety of dual orexin receptor antagonists in primary insomnia: A systematic review and network meta-analysis Sleep Med Rev 2022 61 101573 10.1016/j.smrv.2021.101573 34902823

[28] Zhang L Zhao Z X Objective and subjective measures for sleep disorders Neurosci Bull 2007 23 04 236 240 10.1007/s12264-007-0035-9 17687399 PMC5550587

