# Supplementary material for: Dual orexin receptor antagonists for the treatment of insomnia: systematic review and network meta-analysis
Source: Arq Neuropsiquiatr. 2023 May 31;81(5):475–83. doi: 10.1055/s-0043-1768667 (PMC10232020; doi:10.1055/s-0043-1768667)
Supplement: Supplementary file 1 — Supplementary Material [file 10-1055-s-0043-1768667-s220213.pdf]

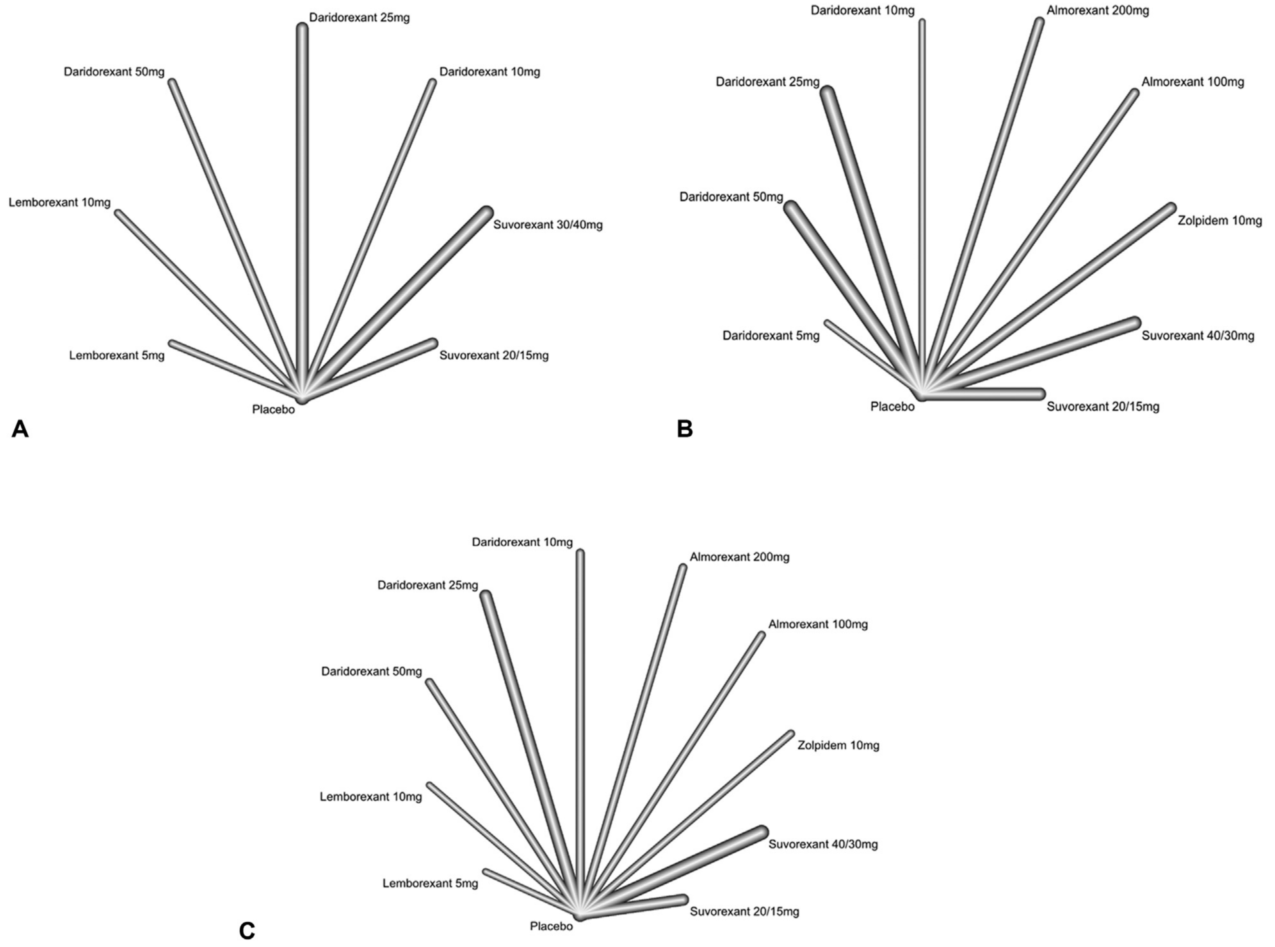


**Supplementary Fig S1** Network graphs. **A.** WASO $\geq$ 3 months; **B.** LPS; **C.** TST.

## Supplementary Table S1

| Certainty assessment | | | | | | | Certainty |
|---|---|---|---|---|---|---|---|
| N° of studies | Study design | Risk of bias | Inconsistency | Indirectness | Imprecision | Other considerations | |
| 8 | randomized trials | not serious | very serious[a] | not serious | not serious | strong association | ⊕⊕⊕◯ Moderate |
| 6 | randomized trials | not serious | very serious[b] | not serious | not serious | strong association | ⊕⊕⊕◯ Moderate |
| 6 | randomized trials | not serious | serious[c] | not serious | not serious | strong association | ⊕⊕⊕⊕ High |
| 8 | randomized trials | not serious | not serious | not serious | not serious | strong association | ⊕⊕⊕⊕ High |

Explanations
a $I^2$ statistic tests revealed very high heterogeneity between studies ($I^2 = 76.1\%$).
b $I^2$ statistic tests revealed very high heterogeneity between studies ($I^2 = 81.4\%$).
c $I^2$ statistic tests revealed high heterogeneity between studies ($I^2 = 56.5\%$).

 © 2023. Academia Brasileira de Neurologia. All rights reserved.
